# Supplementary material for: Pro-Apoptotic and Anti-Cancer Activity of the Vernonanthura Nudiflora Hydroethanolic Extract
Source: Cancers (Basel). 2023 Mar 6;15(5):1627. doi: 10.3390/cancers15051627 (PMC10000589; doi:10.3390/cancers15051627)
Supplement: Supplementary file 1 [file cancers-15-01627-s001.zip › cancers-2086729-supplementary.pdf]

Supplementary data

Regarding Vernonanthura nudiflora, according to our records, it has not been mentioned in any pharmacopoeia up to now. A certain combination of compounds belonging to it are responsible for the effects that will be seen below, so this extract has the potential to become a phytomedicine.

Table S1. Antibodies used in this study

Antibodies against the indicated protein, their catalogue number, source, and the dilutions used in immunoblotting (WB) and immunofluorescence (IF) experiments are listed.

Antibody	Source and Cat. No.	WB	IF
Rabbit polyclonal anti-VDAC1	Abcam, Cambridge, UK, ab15895	1:5000	1:500
Rabbit monoclonal anti-HK-I	Abcam, Cambridge, UK, ab150423	-	1:750
Rabbit monoclonal anti-Nestin	Abcam, Cambridge, UK, ab105389	-	1:750
Rabbit monoclonal anti-Glut-1	Abcam, Cambridge, UK, ab115730	-	1:750
Mouse monoclonal anti-SOX2	Abcam, Cambridge, UK, ab171380	-	1:200
Anti-α-SMA	Abcam, Cambridge, UK, ab5694	-	1:750
Anti-CD31	Abcam, Cambridge, UK, ab28364	-	1:750
Mouse monoclonal anti-GAPDH	Abcam, Cambridge, UK, ab9484	-	1:750
Mouse monoclonal anti-Ki-67	Biolegend, San Diego, California, US, 350502	-	1:750
Donkey anti-mouse-Alexa fluor 488	Abcam, Cambridge, UK, ab150109	-	1:750
Goat anti-rabbit IgG-Alexa fluor 555	Abcam, Cambridge, UK, ab150086	-	1:850
Goat anti-rabbit Alexa fluor 488	Abcam, Cambridge, UK, -ab150078	-	1:750
Goat anti-mouse Alexa fluor 555	Abcam, Cambridge, UK, ab150114	-	1:750
Goat anti-rabbit-HRP	Promega, Wisconsin W4018	1:15,000	-
Donkey anti-mouse-HRP	Abcam, Cambridge, UK, ab98799	1:10,000	-
Mouse monoclonal anti-β-actin	Millipore, Billerica, MA, MAB1501	1:40,000	-

Table S2. The compounds identified in plant extracts – Vern, Bac, Pla using GC-MS analysis

CG-MS analyses of the hydroethanolic extracts of the plants Ver, Bac and Pla were carried out using: a 7890B Mass-Detector; 5977A, Agilent Technologies; Column 5MS UI. The compounds were identified using Library Name W 10N 14L (NIST MS Search 2.2). The various names presenting each compound, the quality of identification (maximum is 100%), and the peak area (Ab*s) are given.

GC/MS identified compounds		
**Plant Vern Extract**	**Plant Bac Extract**	**Plant Extract Pla**
**1**) Hexadecanoic acid ethyl ester (Peak 1) Area (Ab*s)=33,110,310 Quality=99<br><u>Other names</u>: Palmitic acid, ethyl ester; Ethyl hexadecanoate; Ethyl palmitate; Ethyl n-hexadecanoate; Ethyl hexadecanoate	**1**) Hexadecanoic acid, ethyl ester (Peak 26) Area (Ab*s)= 10,220,250<br>Quality=99	**1**) Hexadecanoic acid, ethyl ester (Peak 7) Area (Ab*s)= 50,748,144 Quality=99
**2**) Phytol (Peak 2) Area (Ab*s)=13,850,839 Quality=99	**2**) Phytol (Peak 29) Area (Ab*s)=3,989,191 Quality=93	**2**) Phytol (Peak 8) Area (Ab*s)= 18,787,619 Quality=96
**3**) Linoleic acid ethyl ester (Peak 3) |ETHYL (9z,12z)- 9,12-Octadecadienoate (Ab*s)=20,555,645 Quality=99	**3**) Linoleic acid ethyl ester 9,12-<br><u>Other names</u>: Octadecadienoic acid, ethyl ester (Peak 30) Area (Ab*s)= 5,514,452 Quality=99	**3**) Linoleic acid ethyl ester (Peak 11) Area (Ab*s)= 30,507,168 Quality=99
**4**) 9,12,15-Octadecatrienoic acid ethyl ester, (Z,Z,Z)- (Peak 4) (Ab*s)=23,074,287 Quality=99<br><u>Other names</u>: Linolenic acid, ethyl ester; Ethyl cis,cis,cis-9,12,15-octadecatrienoate; Ethyl linolenate; Ethyl α-linolenate; Ethyl (Z,Z,Z)-	**4**) 9,12,15-Octadecatrienoic acid, ethyl ester, (Z,Z,Z)-<br>(Peak 31) Area (Ab*s)= 5,710,334 Quality=99	**4**) 9,12,15-Octadecatrienoic acid, ethyl ester, (Z,Z,Z)- (Peak 12) Area (Ab*s)= 63,412,281 Quality=99

9,12,15-octadecatrienoate; 9,12,15-Octadecatrienoic acid, ethyl ester, (9Z,12Z,15Z)-; ethyl (9Z,12Z,15Z)-9,12,15-octadecatrienoate		
**5**) Octadecanoic acid ethyl ester (Peak 5) (Ab*s)=3,107,973 Quality=99<br><u>Other names</u>: Stearic acid, ethyl ester; Ethyl n-octadecanoate; Ethyl octadecanoate; Ethyl stearate; Radia 7185; Ethyl ocatadecanoate; Ethyl octadecanoate (Ethyl stearate)	**5**) Benzene, 1-methoxy-2-(1-methylethenyl)-	**5**) Octadecanoic acid, ethyl ester (Peak 13) Area (Ab*s)= 4,630,724,Quality=99<br><u>Other names</u>: Stearic acid, ethyl ester; Ethyl n-octadecanoate; Ethyl octadecanoate; Ethyl stearate; Radia 7185; Ethyl ocatadecanoate; Ethyl octadecanoate (Ethyl stearate)
**6**) 1,2- Benzenedicarboxylic acid, bit (2-ethylhexyl) ester (Peak 6)<br><u>Other names</u>: Phthalic acid, diisobutyl ester; Diisobutyl phthalate; Hexaplas M/1B; Isobutyl phthalate; Palatinol IC; DIBP; Diisobutylester kyseliny ftalove; Kodaflex DIBP; 1,2-Benzenedicarboxylic acid, di(2-methylpropyl) ester; 1,2-Benzenedicarboxylic acid, 1,2-bis(2-methylpropyl) ester; Bis(2-methylpropyl) phthalate; NSC 15316; Isobutyl-o-phthalate; di-2-methylpropyl phthalate	**6**) 4H -Pyran-4-one, 2,3-dihydro-3,5-dihydroxy-6-methyl- (Peak 3)) Area (Ab*s)= 24,981,669 Quality=96	**6**) 4H-Pyran-4-one, 2,3-dihydro-3,5-dihydroxy-6-methyl- (Peak 2)) Area (Ab*s)= 27,382,476 Quality=95
**7**) 2,5 Dimethyldiphenylsulfone<br><u>Other names</u>: Thiophene, 2,5-dihydro-, 1,1-dioxide; 2,5-Dihydrothiophene 1,1-dioxide; 3-Sulfolene; Butadiene sulfone; Sulfolene; NCI-C04557; 2,5-Dihydrothiophene dioxide; Sulfol-3-ene; β-Sulfolene; 2,5-Dihydrothiophene S,S-dioxide; NSC 48532; Sulpholene	**7**) 2-Methoxy- 4-vinylphenol (Peak 10)<br>Area (Ab*s)= 7,305,730<br>Quality=97	**7**) 9,12,15-Octadecatrienoic acid, (Z,Z,Z)- (Peak 10) Area (Ab*s)= 47,918,691 Quality=99
**8**) Stigmasta-5,22-dien-3-ol (Peak 11) Area (Ab*s)= 11,567,27423.	**8**) 4-Methyleneisophorone (peak 6) Area (Ab*s)= 21,458,921 Quality=97	**8**) 2-Methoxy-4- vinylphenol (Peak 3)) Area (Ab*s)= 3,985,200 Quality=94
**9**) OLEAN-12-EN-3-OL(Peak 8) (Ab*s)= 143,738,307 Quality=93<br><u>Other names</u>: Beta Amyrin 3-acetate 3,beta.-Acetoxyolean-12-ene Olean-12-en-3-ol, acetate, (3.beta.	**9**) 1H-Cycloprop[e]azulen-7-ol, decahydro-1,1,7-trimethyl-4-methylene-, [1ar (1a.alpha., 4a.alpha.,7.beta.,7a.beta.,7b.alpha.)]-(Peak 18) Area (Ab*s)= 13,447,105 Quality=99	**9**) 9,12-Octadecadienoic acid (Z,Z)- (Peak 9) Area (Ab*s)=8,205,041 Quality=99
**10**) URS-20(30) –EN- 3-OL	**10**) 1,1,4,7 tetramethyldecahydro-1h-cyclopropa[e]azulen-4-OL, (Peak 19) Area (Ab*s)= 60,683,772 Quality=97	10) n-Hexadecanoic acid (Peak 6) Area (Ab*s) = 25,158,853 Quality=93
**11**) (3.beta.,21.beta.) –A'- neogammacer-22(29)-en-3-ol (Peak 10) Area (Ab*s)= 59,020,551 Quality=93	**11**) Ledol	**11**) Hexadecanoic acid, 2-hydroxy-1 –(hydroxymethyl) ethyl ester
**12**) Pentacyclic Triterpene – Alcohol (Peak 11) Area (Ab*s)= 22,398,502 Quality=93	**12**) 4aH-Cycloprop[e]azulen-4a-ol, decahydro-1,1,4,7-tetramethyl-, [1aR (1a.alpha., 4.beta.,4a.beta., 7.alpha.,7a.beta.,7b.alpha.)]-(Peak 17) Area (Ab*s)= 30,609,427, Quality=99	**12**)1,3,12- Nonadecatriene
	**13**) 2(10)-Pinene (Peak 2)) Area (Ab*s)= 5,807,189 Quality=95	**13**) gamma. – Sitosterol (Peak 22) Area (Ab*s)= 35,635,132
	**14**) Trans-Sinapyl alcohol (Peak 27) Area (Ab*s)= 5,523,775 Quality=96	
	**15**) 2-Naphthalenemethanol, decahydro-alpha,alpha.,4a-trimethyl-8-methylene-, [2R-(2.alpha.,4a.alpha.,8a.beta.)]-(Peak 23) Area (Ab*s)=	

	26,189,433 Quality=97	
	**16**) 4-oxo-adamantane-1-carboxylic acid methyl ester Pyrene, 1,2,3,6,7,8-hexahydro- (Peak 28) Area (Ab*s)= 28,617,055 Quality=90	
	**17)** 2-Hydroxy-3,5,5-trimethyl-cyclohex-2-enone, (peak 4) Area (Ab*s)= 10,619,964 Quality=96	
	18) 3-Cyclohexen-1-ol,5-methylene-6-(1-methylethenyl)- (peak 5)) Area (Ab*s)= 34,153,434 Quality=91	.
	**19)** (4H)1-Benzopyran-4-one, 3,5,6,7-tetrahydroxy-8-methyl-2-phenyl<br>Other names: Platanin	
	**20**) Valerena-4,7(11)-diene (peak 15 Area (Ab*s)= 6,007,4363 Quality=96	

Table S3. Vern plant extract cell death activity is stable after heating the extract

Vern plant extract was incubated 10 min at the indicated temperature and then assayed for cell death induction following 24 h incubation of SH-SY5Y cells with the indicated dilution of the treated extract.

**Tempe. ^{o}C**		**Cell death, %**		
	**Ver plant extract dilution**	**1:2000**	**1:1000**	**1:500**
4		37	83	98
45		-	84	98
60		40	91	98
80		57	92	99

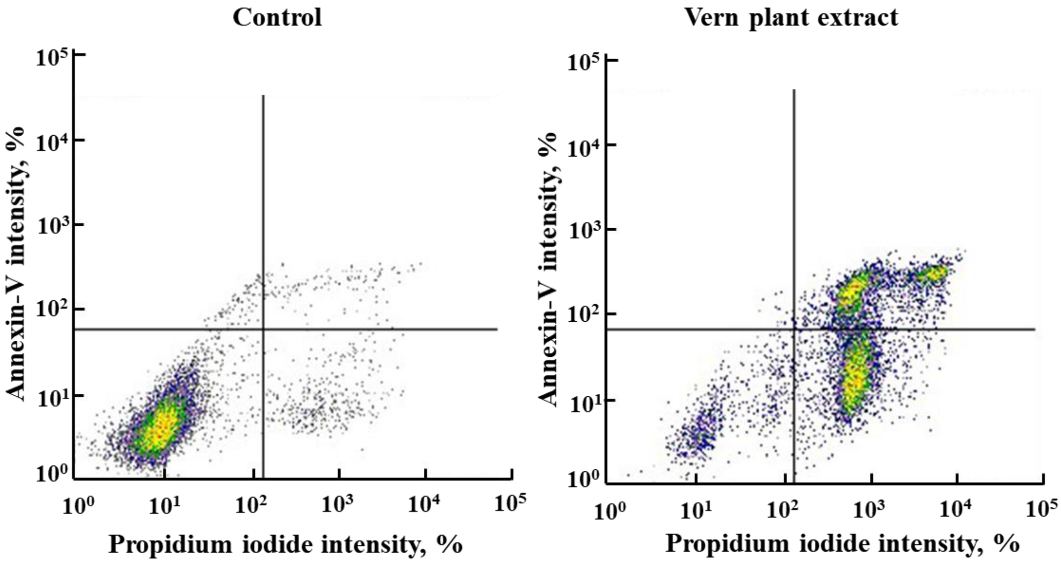

Figure S1. Apoptosis analysis using Annexin V and PI and FACS analysis

SH-SY5Y cells were incubated (24 h) with Vern plant extract (1:250), and then analyzed for apoptosis using Annexin V/PI staining and FACS, representative FACS results are shown.

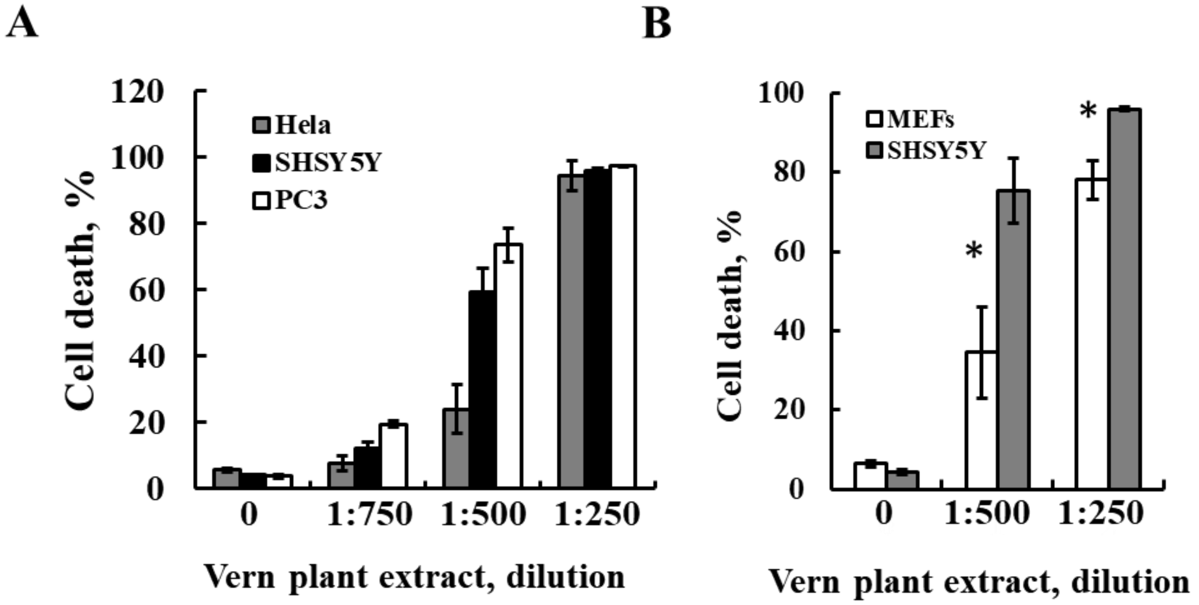

Figure S2. Vern plant extract effect on different cell lines

SH-SY5Y, Hela and PC-3 cells (A) or SH-SY5Y and MEFs cells (B) were incubated for 24h with the indicated dilutions of plant Vern extract, and cell death was analyzed by Pi staining and FACS analysis (n=3). Results are means ± SEM. **P*<0.01.

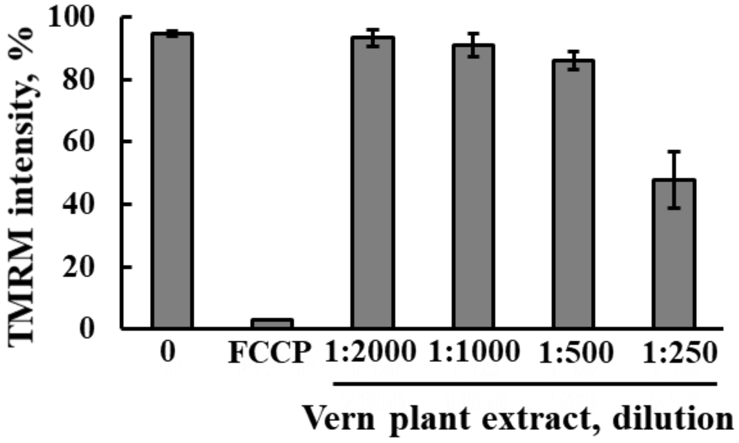

Figure S3. Vern plant extract effect on mitochondrial membrane potential

SH-SY5Y cells were incubated for 24h with the indicated dilutions of plant Vern extract, or FCCP (50 μM), harvested and incubated with TMRM (400nM, 20min) and analyzed for mitochondrial membrane potential ($\Delta\Psi$) by flow cytometer. Results are present as TMRM fluorescence intensity as a percentage of control (n=3). Results are means ± SEM.

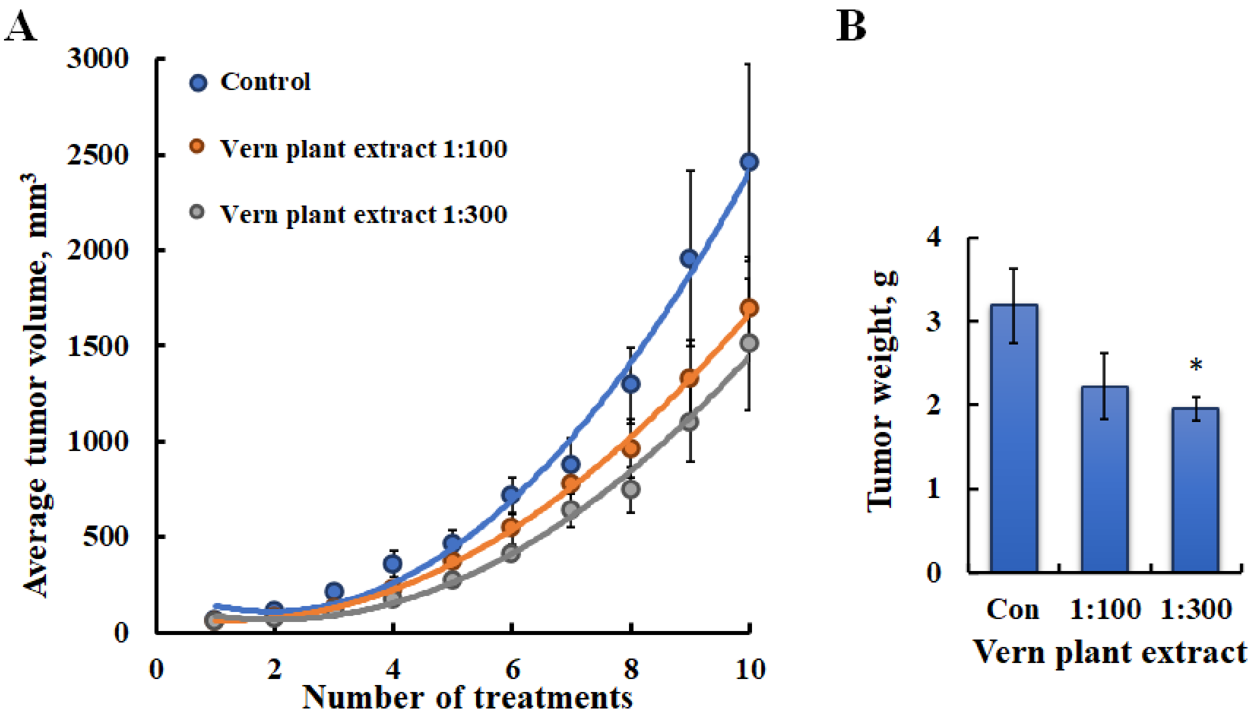

Figure S4. Inhibition of tumor development by plant A extract in glioblastoma xenograft mouse model
U-87MG cells (1.8 × 10^6 cells/mouse) were S.C. inoculated into nude mice. Tumor volume was monitored (using a digital caliper) and on day 14, when the tumor volume was between 40 and 60 mm^3, the mice were divided into three groups with a similar average volume calculated per group (5 or 6 mice per group). The three mice groups were subjected to the following treatments: control (ethanol to a final concentration 0.14%) or Vern plant extract to a final dilution of 1:100 or 1:300, calculated according to the tumor volume. (**A**) The calculated average tumor volumes as a function of time are presented as means ± SEM (n=5 or 6 mice). (**B**) The calculated average tumor weights are presented as means ± SEM. *P<0.05.
